# Association between diabetes of different durations and hip fracture in middle-aged and older people: a stratified cohort study from CHARLS 2011–2020

**DOI:** 10.1186/s12889-025-21923-0

**Published:** 2025-02-19

**Authors:** Yu Chang, Yunda Huang, Ruonan Li, Li Gui

**Affiliations:** 1 Department of Endocrinology, The Third People’s Hospital of Yunnan Province, The Second Affiliated Hospital of Dali University, 292 Beijing Road, Kunming, Yunnan, 650011 China; 2 Department of Geriatrics, The Third People’s Hospital of Yunnan Province, The Second Affiliated Hospital of Dali University, Kunming, China

**Keywords:** Diabetes, Diabetes durations, Hip fracture, CHARLS

## Abstract

**Background:**

The effect of the duration of diabetes on hip fracture is inconsistent. The aim of this study was to analyze the association between different durations of diabetes and hip fracture.

**Methods:**

This cohort study included participants from the China Health and Retirement Longitudinal Study (CHARLS) 2011–2020. Diabetes was defined as glycated hemoglobin A1c ≥ 6.5%, fasting blood glucose ≥ 126 mg/dL, random blood glucose ≥ 200 mg/dL, or previous diagnosis of diabetes. Participants were stratified according to diabetes duration, and information was collected on their first hip fracture. The association between diabetes of different durations and hip fracture was assessed using Cox proportional risk models and Kaplan-Meier curves.

**Results:**

A total of 9,927 participants with a mean age of 58.4 ± 8.7 and 54.3% female were included, and 574 participants suffered a hip fracture. Compared with no diabetes, the associations between overall diabetes, newly diagnosed diabetes, diabetes with a duration of < 6 years, and hip fracture were all not significant, all *P* > 0.05. Known diabetes and diabetes of duration ≥ 6 years significantly increased the risk of hip fracture, with hazard ratios (HRs) and 95% confidence intervals (CIs) of 1.69 (1.19 ~ 2.4), *P* = 0.003, and 2.2 (1.34 ~ 3.61), *P* = 0.002.

**Conclusions:**

Neither newly diagnosed diabetes nor diabetes with a disease duration of < 6 years was associated with hip fracture compared with no diabetes people. When the duration of diabetes is ≥ 6 years, the risk of hip fracture is significantly increased, and appropriate preventive measures are recommended.

## Introduction

Hip fracture is a common trauma in middle-aged and elderly people and has become an important public health problem. The number of hip fractures is projected to increase from 1.66 million in 1990 to 6.26 million in 2050. Although Europe and North America accounted for half of all hip fractures worldwide in 1990, this proportion is expected to decline to one quarter by 2050, with the proportion of hip fractures in Asia rising to 50 per cent as the Asian population grows in size and ageing increases [1]. The number of new hip fracture cases in China is more than 1 million per year [2]. Hip fracture has a high disability and mortality rate and is therefore also known as the “last fracture of life.” Although the mortality rate of hip fracture has decreased in recent decades due to advances in surgical treatment, the mortality rate is still as high as 20% at 1 year after fracture, and 20–60% of survivors still require long-term home care [3–5]. Hip fractures also carry a significant financial burden, with the average cost of initial hospitalization being approximately US$9,500 and subsequent rehabilitation care costing US$19,000–66,000 [6, 7]. Therefore, early detection of risk factors for hip fracture and development of preventive measures for hip fracture are essential.

Diabetes is the 8th leading cause of death and disability, affecting all people worldwide [8]. According to an analysis of the Global Burden of Diseases, Injuries, and Risk Factors Study [9], the number of people with diabetes worldwide in 2021 was already as high as 529 million. The overall global age-standardized prevalence of diabetes is 6.1%, with a prevalence of more than 15% in middle-aged and older adults aged ≥ 45 years [9]. The dangers of diabetes have been well documented, as it significantly affects the cardiovascular, renal, neurological, and ophthalmic systems of patients [10, 11]. Patients with diabetes have a six-fold increase in all-cause mortality compared to those without diabetes [12]. Diabetes is a chronic disease with lasting health effects and a higher prevalence than hip fractures, so the impact of diabetes on the health of society cannot be ignored [9, 13]. Studies have also shown that diabetes increases overall fracture risk by 20% to 3-fold [14–17], particularly hip fracture risk [18, 19]. This risk appears to be related to the duration of diabetes, with Koh WP et al. finding a 40% increased risk of hip fracture when the duration of diabetes was less than 5 years, and a 166% increased risk of hip fracture when the duration of diabetes was ≥ 15 years [20]. In a study by Melton et al. the risk of hip fracture was found to be unincreased when the duration of diabetes was < 10 years [21]. In a prospective cohort study of women by Janghorbani M et al., the relative risk of hip fracture was increased by 70%, 80%, and 210% in those with diabetes duration < 5 years, 5–10 years, and ≥ 11 years, respectively [22]. No large population-based studies have been reported in this area in China.

As the risk of hip fracture in populations with different diabetes durations is inconsistent and there is a lack of studies in Chinese community-based populations, we designed this cohort study. We included participants from the China Health and Retirement Longitudinal Study (CHARLS) 2011–2020, stratified according to the duration of diabetes, and created 3 stratified cohorts. The aim was to analyze the association between diabetes of different durations and hip fracture in a community-based population of middle-aged and older Chinese adults.

## Methods

### Study population

Participants in this cohort study were drawn from the CHARLS, a nationally representative cohort study from China. The baseline survey was conducted in 2011 and subsequently followed up every 2–3 years, collecting a wide range of health and socioeconomic information on the middle-aged and elderly population aged 45 years or older in the Chinese community. The CHARLS study was approved by the Biomedical Ethics Committee of Peking University (approval number: IRB00001052-11015), about whom detailed information has been reported previously [23].

**Fig. 1:**
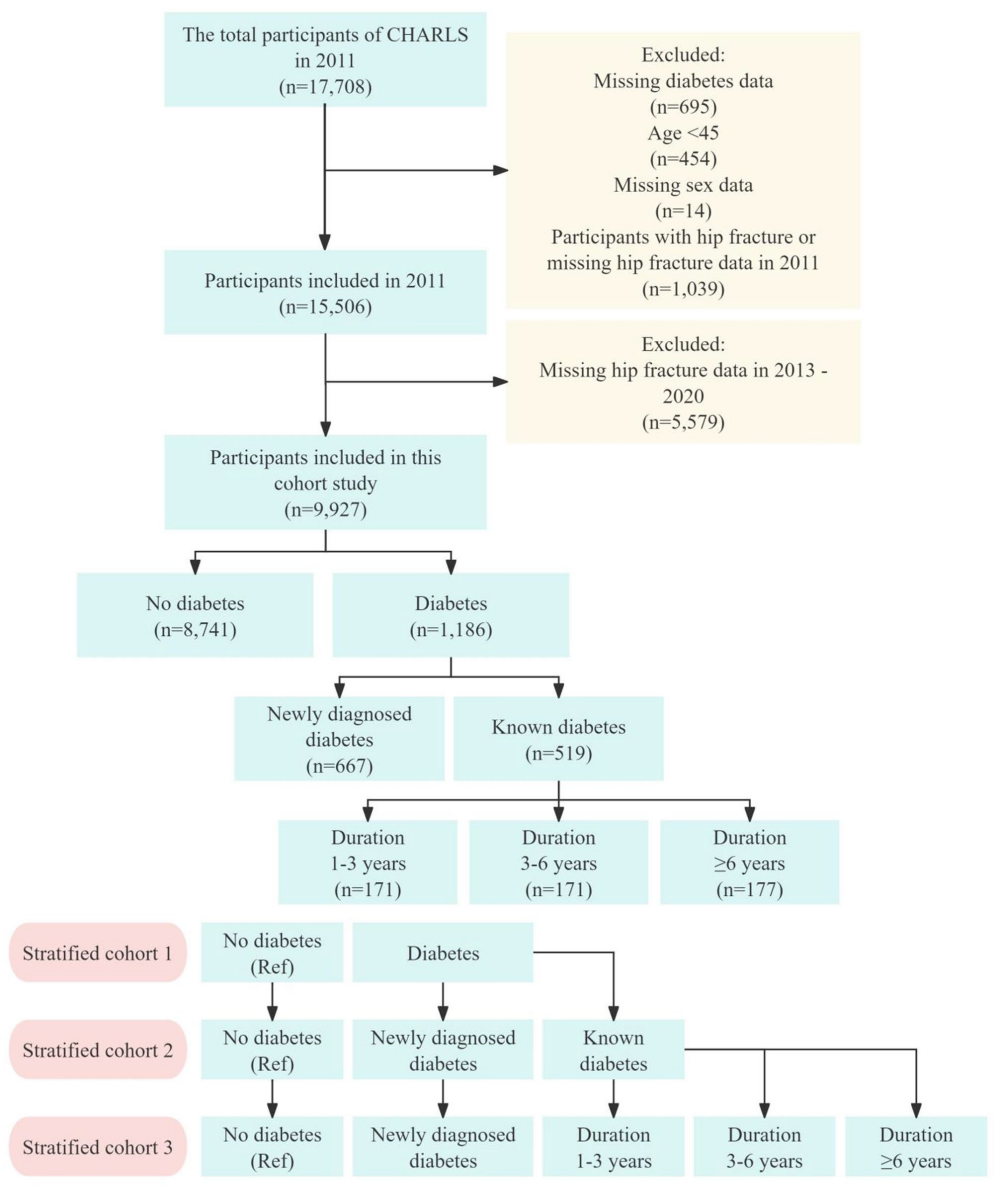
Flow chart of the screening of participants

We included participants from the 2011 baseline survey in CHARLS, and the following groups were excluded: (1) missing information on diabetes (*n* = 695); (2) age < 45 years (*n* = 454); (3) missing information on gender (*n* = 14); (4) with a previous hip fracture or missing information on a hip fracture (*n* = 1,039); a total of 15,506 participants were enrolled. Follow-up visits were conducted in 2013, 2015, 2018, and 2020, respectively. After further exclusion of people lost to follow-up or missing information on hip fracture (*n* = 5,579), a total of 9,927 participants were ultimately enrolled in this study, including 8,741 participants without diabetes and 1,186 with diabetes. We further categorized participants with diabetes by disease duration: newly diagnosed diabetes (*n* = 667), known diabetes (*n* = 519), diabetes duration of 1–3 years (*n* = 171), 3–6 years (*n* = 171), and ≥ 6 years (*n* = 177) (Fig. 1).

The study was conducted in accordance with the Strengthening the Reporting of Observational Studies in Epidemiology (STROBE) guideline recommendations related to cohort studies [24].

### Assessment of diabetes and its duration

We used two methods to determine whether participants had diabetes at baseline: past medical history and blood test results. Past medical history was based on the questions in the questionnaire, “Have you been diagnosed with diabetes by a doctor?” and “When was the condition first diagnosed or known by yourself?” Participants were defined as having a known diabetes when they answered yes or when the duration of illness was not missing, and the duration of diabetes was also determined based on the questions above.

For participants who had not been previously found to have diabetes, we further determined the presence of diabetes based on blood test results. According to the standard protocol, respondents were asked to fast overnight, and venous blood was collected by medically trained staff at the Chinese Center for Disease Control and Prevention for fasting blood glucose and glycosylated hemoglobin. If the respondent did not fast strictly, we marked the blood glucose test results as randomized blood glucose. According to the diagnostic criteria published by the American Diabetes Association in 2024 [25], participants were defined as having a newly diagnosed diabetes when one of the following was present: (1) glycated hemoglobin A1c (HbA1c) ≥ 6.5%, (2) fasting blood glucose ≥ 126 mg/dL, or (3) random blood glucose ≥ 200 mg/dL. Because an oral glucose tolerance test (OGTT) was not performed in CHARLS, we did not use diagnostic criteria based on 2-hour post-OGTT blood glucose results.

### Assessment of hip fracture

Participants’ hip fracture information was derived from two questions in the questionnaire: at the baseline survey, “Have you ever fractured your hip?” and at follow-up in 2013, 2015, 2018, and 2020, “Have you fractured your hip since we talked in the last interview?”. Since the time to onset of hip fracture was not documented in the CHARLS study, we defined the time variable as the median time from the last follow-up without a fracture to the follow-up when the fracture was first found.

### Covariates

We included information on participants’ demographic characteristics, lifestyle habits, and chronic disease status as covariates. Specifically, demographic information includes gender, age, education level, marital status, and place of residence. Lifestyle habits included smoking status and alcohol consumption. Participants’ smoking status was categorized as never smoked, former smoker, and current smoker; alcohol intake was categorized as never or rarely, less than once a month, and more than once a month. Chronic disease status included hypertension, dyslipidemia, chronic lung diseases, liver disease, heart problems, stroke, kidney disease, and arthritis. Participants’ chronic disease information was determined by the question in the questionnaire “Have you been diagnosed with this chronic disease by a doctor? When the participant answered “Yes”, they were defined as having this chronic disease. The latter information was obtained from the CHARLS 2011 baseline questionnaire.

### Statistical analysis

To explore the association between duration of diabetes and hip fracture, we categorized participants according to when they were diagnosed with diabetes and created 3 cohorts based on time of diagnosis. Specifically, in cohort 1, all participants were categorized as no diabetes and diabetes. In cohort 2, we further categorized participants with diabetes, and all participants were categorized as no diabetes, newly diagnosed diabetes, and known diabetes. In Cohort 3, we further categorized known diabetes according to duration, and to have similar sample sizes in each group, we used 3 and 6 years as cut-off points, whereby all participants were categorized as no diabetes, newly diagnosed diabetes, diabetes with a duration of 1–3 years, a duration of 3–6 years, and a duration of ≥ 6 years.

Continuous variables are represented by mean ± standard deviation (SD) for normal distributions, median and interquartile range (IQR) for skewed distributions, and categorical variables are expressed as counts and percentages. The baseline characteristics of participants in Cohort 3 in 2011 were compared using the chi-square test, ANOVA, and Kruskal-Wallis tests. Univariate and multivariate Cox proportional risk models were used to estimate hazard ratios (HRs) and 95% confidence intervals (CI) for the association between diabetes and its duration with new hip fractures. The following covariates were adjusted in the multifactorial analysis: gender, age, education, marital, residence, smoking status, alcohol consumption, hypertension, dyslipidemia, chronic lung diseases, liver disease, heart problems, stroke, kidney disease, and arthritis. To further understand whether there was a linear trend between diabetes duration and the risk of hip fracture, we used a trend test in cohort 3. In addition, we used Kaplan-Meier curves to demonstrate cumulative hip fracture events over time in the different cohorts, as well as a log-rank test to assess differences between groups of participants with different diabetes durations.

Considering that hip fracture in participants may be directly caused by a traffic accident or any major accidental injury not related to diabetes mellitus, a sensitivity analysis was performed to verify the stability of the results. After excluding participants who had been involved in a traffic accident or any major accidental injury, we analyzed the association between diabetes and hip fracture again in all cohorts.

All the analyses were performed with the statistical software Stata/MP version 17.0 (StataCorp LP, College Station, TX, USA) and Free Statistics software version 1.9.2 (Beijing Free Clinical Medical Technology Co., Ltd.). The level of statistical significance was set at *p* < 0.05 (two-sided).

## Results

### Baseline characteristics of study participants

Table 1 shows the baseline characteristics of the participants in cohort 3 in 2011. Compared with participants with no diabetes and diabetes with a duration of < 6 years, participants with a diabetes duration of ≥ 6 years were the oldest, had the highest proportion of education and living in a town, the highest alcohol intake, and the highest proportions of heart disease, stroke, and kidney disease, as well as they had the highest mean HbA1c and blood glucose. Compared with participants in the other 4 groups, participants with diabetes duration of 3–6 years had the highest rates of hypertension and dyslipidemia; participants with diabetes duration of 1–3 years had the highest rates of being female and having liver disease. Notably, participants with newly diagnosed diabetes had the lowest level of education and the highest percentage living in rural areas compared to those without diabetes and those with known diabetes.

**Table 1:** The baseline characteristics of participants were categorized according to different durations of diabetes

	Total (*n* = 9927)	No diabetes (*n* = 8741)	Newly diagnosed diabetes (*n* = 667)	Diabetes duration 1–3 years (*n* = 171)	Diabetes duration 3–6 years (*n* = 171)	Diabetes duration ≥ 6 years (*n* = 177)	*P*-value
Age, year, mean ± SD	58.4 ± 8.7	58.3 ± 8.7	59.5 ± 8.9	59.2 ± 8.4	58.0 ± 7.6	60.9 ± 8.0	< 0.001
Gender, n (%)							0.027
Female	5392 (54.3)	4742 (54.3)	349 (52.3)	113 (66.1)	95 (55.6)	93 (52.5)	
Male	4535 (45.7)	3999 (45.7)	318 (47.7)	58 (33.9)	76 (44.4)	84 (47.5)	
Education, n (%)							0.029
Primary school or below	6793 (68.4)	5998 (68.6)	464 (69.6)	115 (67.3)	110 (64.3)	106 (59.9)	
Middle school	2040 (20.6)	1795 (20.5)	138 (20.7)	34 (19.9)	36 (21.1)	37 (20.9)	
High school or above	1094 (11.0)	948 (10.8)	65 (9.7)	22 (12.9)	25 (14.6)	34 (19.2)	
Marital, n (%)							0.22
Other	965 ( 9.7)	859 (9.8)	64 (9.6)	18 (10.5)	16 (9.4)	8 (4.5)	
Married	8962 (90.3)	7882 (90.2)	603 (90.4)	153 (89.5)	155 (90.6)	169 (95.5)	
Residence, n (%)							< 0.001
Urban areas	1669 (16.8)	1419 (16.3)	94 (14.1)	39 (22.8)	54 (31.6)	63 (35.6)	
Rural areas	8246 (83.2)	7311 (83.7)	572 (85.9)	132 (77.2)	117 (68.4)	114 (64.4)	
Smoking status, n (%)							< 0.001
Never smoked	6180 (62.3)	5437 (62.2)	395 (59.2)	121 (70.8)	116 (67.8)	111 (62.7)	
Former smoker	762 ( 7.7)	642 (7.3)	56 (8.4)	18 (10.5)	23 (13.5)	23 (13)	
Current smoker	2982 (30.0)	2659 (30.4)	216 (32.4)	32 (18.7)	32 (18.7)	43 (24.3)	
Alcohol consumption, n (%)							0.001
Never or rarely	6135 (81.7)	5428 (82.3)	381 (79.4)	114 (78.6)	105 (77.8)	107 (72.3)	
Less than once a month	777 (10.4)	675 (10.2)	54 (11.2)	16 (11)	17 (12.6)	15 (10.1)	
More than once a month	593 ( 7.9)	494 (7.5)	45 (9.4)	15 (10.3)	13 (9.6)	26 (17.6)	
Hypertension, n (%)	2263 (22.9)	1792 (20.6)	201 (30.3)	87 (50.9)	92 (53.8)	91 (51.4)	< 0.001
Dyslipidemia, n (%)	887 ( 9.1)	623 (7.3)	72 (11)	59 (34.7)	69 (40.6)	64 (36.8)	< 0.001
Chronic lung diseases, n (%)	909 ( 9.2)	795 (9.1)	58 (8.7)	24 (14)	16 (9.4)	16 (9)	0.283
Liver disease, n (%)	382 ( 3.9)	320 (3.7)	25 (3.8)	13 (7.6)	11 (6.5)	13 (7.4)	0.003
Heart problems, n (%)	1083 (11.0)	891 (10.2)	72 (10.8)	30 (17.6)	39 (23.1)	51 (29)	< 0.001
Stroke, n (%)	170 ( 1.7)	128 (1.5)	14 (2.1)	6 (3.5)	7 (4.1)	15 (8.5)	< 0.001
Kidney disease, n (%)	612 ( 6.2)	521 (6)	40 (6)	12 (7.1)	18 (10.5)	21 (11.9)	0.003
Digestive disease, n (%)	2342 (23.7)	2078 (23.8)	143 (21.5)	47 (27.5)	37 (21.6)	37 (20.9)	0.348
Arthritis, n (%)	3460 (34.9)	3026 (34.7)	242 (36.3)	64 (37.6)	62 (36.3)	66 (37.3)	0.759
HbA1c, %, mean ± SD	5.2 ± 0.8	5.1 ± 0.4	6.0 ± 1.4	5.9 ± 1.3	6.5 ± 1.8	6.8 ± 1.8	< 0.001
HbA1c, mmol/mol, mean ± SD	33.9 ± 8.4	32.2 ± 4.3	42.1 ± 15.0	41.2 ± 14.6	47.9 ± 19.6	51.0 ± 19.9	< 0.001
FBG, mg/dl, median (IQR)	101.9 (94.3, 112.0)	100.1 (93.4, 107.3)	140.9 (130.1, 168.1)	115.9 (101.9, 139.1)	131.2 (103.2, 187.3)	144.4 (108.4, 202.7)	< 0.001
RBG, mg/dl, median (IQR)	106.9 (93.5, 128.9)	105.7 (92.3, 124.4)	234.0 (216.2, 301.5)	166.6 (116.6, 356.2)	132.3 (114.5, 155.3)	167.6 (133.4, 227.9)	< 0.001

Primary school or below, ≤ 6 years of education; Middle school, 7–9 years of education; High school or above, ≥ 10 years of education

Abbreviations: HbA1c, glycated hemoglobin A1c; FBG, fasting blood glucose; RBG, random blood glucose

### Association between diabetes of different durations and hip fracture

After 9 years of follow-up, 574 participants experienced a hip fracture, with an annual incidence rate of 642/100,000. Table 2 shows the results of the univariate and multivariate Cox proportional risk models for all 3 cohorts, with the reference group set to be no diabetes. The overall results of the univariate and multivariate analyses were similar.

**Table 2:** Association between diabetes of different durations and hip fracture

Stratified cohorts	*n*. total	Cases (%)	Univariate analysis			Multiplicity analysis	
			HR (95% CI)	P-value		HR (95% CI)	P-value
Cohort 1							
No diabetes	8741	493 (5.6)	1(Ref)			1(Ref)	
Diabetes	1186	81 (6.8)	1.22 (0.97 ~ 1.55)	0.094		1.24 (0.94 ~ 1.63)	0.128
Cohort 2							
No diabetes	8741	493 (5.6)	1(Ref)			1(Ref)	
Newly diagnosed diabetes	667	38 (5.7)	1.01 (0.73 ~ 1.41)	0.933		0.89 (0.59 ~ 1.34)	0.583
Known diabetes	519	43 (8.3)	1.49 (1.09 ~ 2.04)	0.012		1.69 (1.19 ~ 2.4)	0.003
Cohort 3							
No diabetes	8741	493 (5.6)	1(Ref)			1(Ref)	
Newly diagnosed diabetes	667	38 (5.7)	1.01 (0.73 ~ 1.41)	0.933		0.89 (0.59 ~ 1.34)	0.583
Duration 1–3 years	171	11 (6.4)	1.15 (0.63 ~ 2.09)	0.652		1.45 (0.79 ~ 2.66)	0.232
Duration 3–6 years	171	11 (6.4)	1.15 (0.63 ~ 2.09)	0.652		1.39 (0.74 ~ 2.64)	0.308
Duration ≥ 6 years	177	21 (11.9)	2.18 (1.41 ~ 3.38)	< 0.001		2.2 (1.34 ~ 3.61)	0.002
Trend.test	9927	574 (5.8)	1.15 (1.05 ~ 1.26)	0.004		1.17 (1.05 ~ 1.31)	0.003

Multiplicity analysis: adjusted for gender, age, education, marital, residence, smoke, drink, hypertension, dyslipidemia, chronic lung diseases, liver disease, heart problems, stroke, kidney disease, and arthritis

Abbreviations: HR, hazards ratio; CI, confidence interval

In cohort 1, we did not further group those with diabetes by duration, compared with participants with no diabetes, diabetes participants did not have an overall increased risk of hip fracture, with an HR and 95% CI of 1.24 (0.94 ~ 1.63) on multivariate analysis, *P* = 0.128.

In cohort 2, we grouped participants with diabetes into newly diagnosed diabetes and known diabetes. Compared with participants with no diabetes, participants with newly diagnosed diabetes did not have an increased risk of hip fracture, with an HR and 95% CI of 0.89 (0.59 ~ 1.34) on multivariate analysis, *P* = 0.583. Participants with known diabetes had a significant 69% increased risk of overall hip fracture, with an HR and 95% CI of 1.69 (1.19 ~ 2.4) on multivariate analysis, *P* = 0.003.

In cohort 3, we divided known diabetes into 3 groups based on duration. Compared with participants with no diabetes, participants with newly diagnosed diabetes did not have an increased risk of hip fracture. We found differences in the risk of hip fracture in diabetes groups with different durations. Compared with participants with no diabetes, participants with diabetes duration of 1–3 and 3–6 years had no significantly increased risk of hip fracture, with HRs and 95% CIs of 1.45 (0.79 ~ 2.66), *P* = 0.232, and 1.39 (0.74 ~ 2.64), *P* = 0.308, respectively, for the multifactorial analysis. Participants with ≥ 6 years of diabetes duration had a significant 120% increased risk of hip fracture, with HR and 95% CI of 2.2 (1.34 ~ 3.61) on multivariate analysis, *P* = 0.002. The HR and 95% CI for the trend test for cohort 3 were 1.17 (1.05 ~ 1.31), *P* = 0.003.

**Fig. 2:**
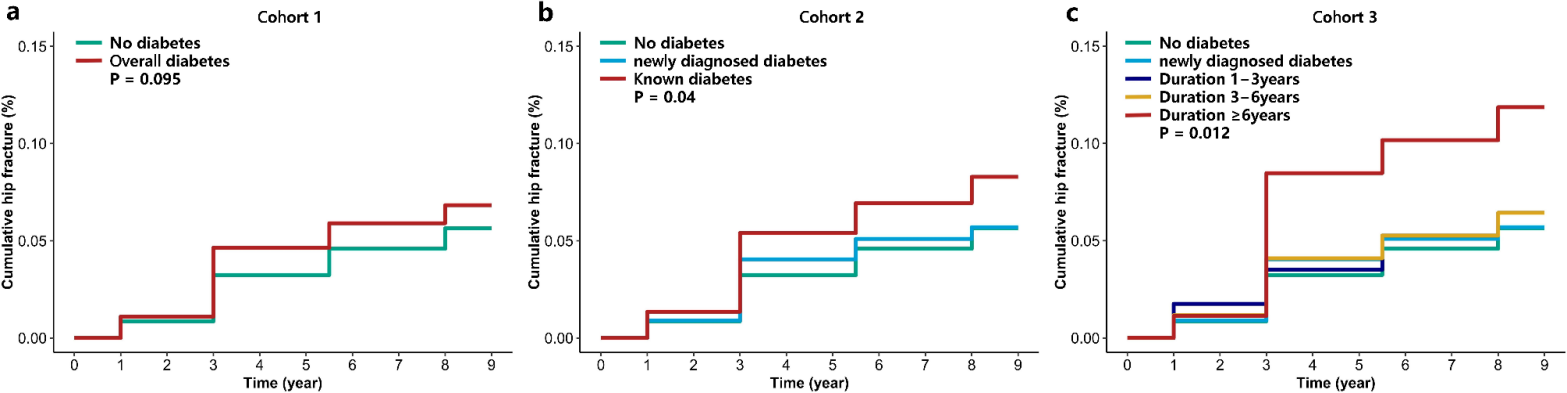
**a**, **b**, and **c** represent the Kaplan-Meier cumulative curves for Cohort 1, Cohort 2, and Cohort 3, respectively

Figure 2 shows the Kaplan-Meier cumulative curves for the 3 cohorts. Figure 2a shows that there was no increase in cumulative hip fracture events in the overall diabetes group, P for log-rank test = 0.095. Figure 2b shows that cumulative hip fracture may have been higher in the group with known diabetes, P for log-rank test = 0.04. Figure 2c shows that cumulative hip fracture may have been highest in the group with diabetes duration of ≥ 6 years, whereas cumulative events in the other groups may have been similar, P for log-rank test = 0.012.

### Subgroup analysis

**Fig. 3:**
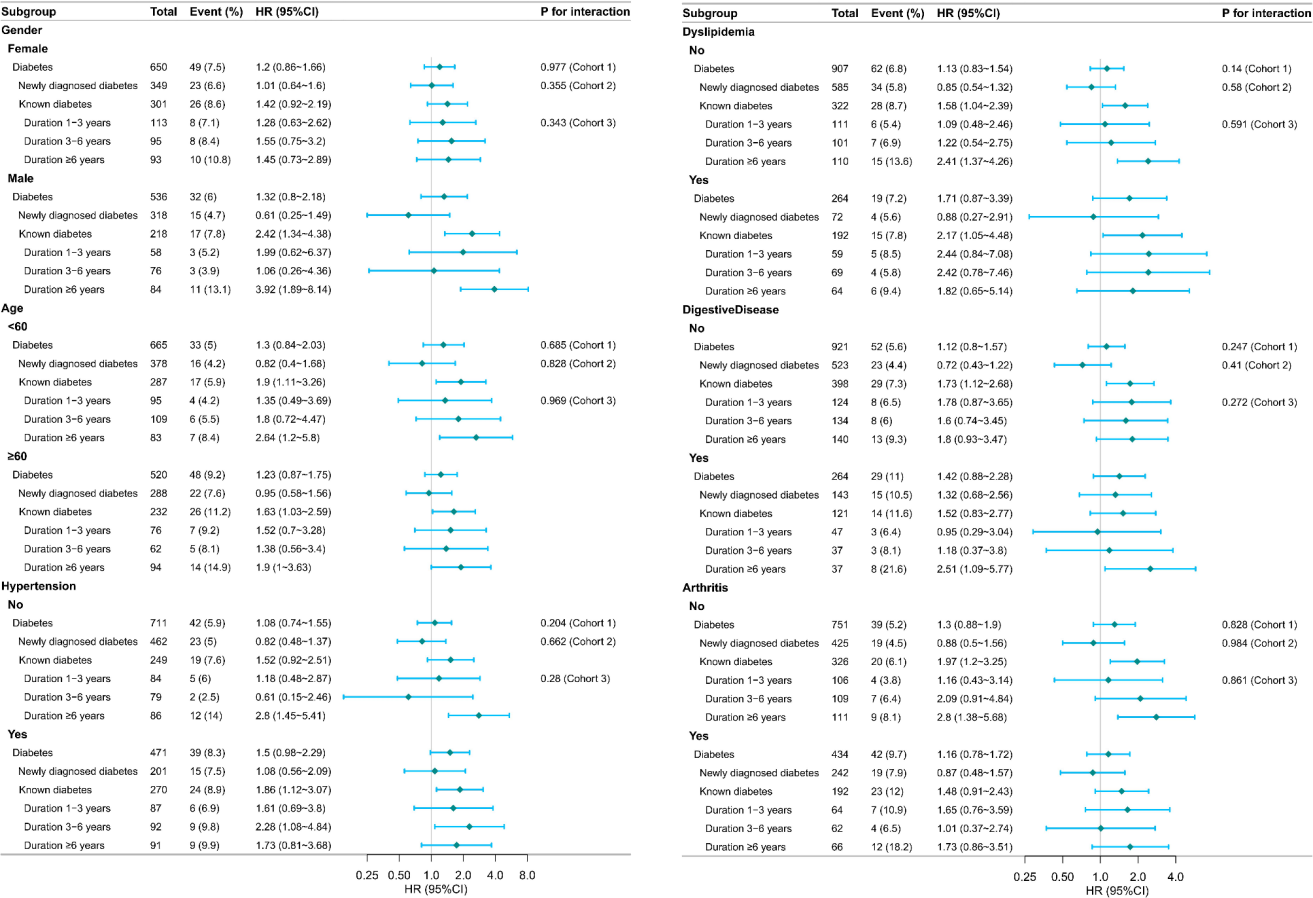
Forest plot of subgroups for multifactorial analysis; the reference groups were set as no diabetes. Subgroups according to gender, age (cut-off point 60 years), hypertension, dyslipidaemia, digestive diseases, and arthritis; all P for interaction > 0.05. Adjusted for gender, age, education, marital, residence, smoke, drink, hypertension, dyslipidemia, chronic lung diseases, liver disease, heart problems, stroke, kidney disease, and arthritis

We grouped the cohorts according to gender, age (cut-off point 60 years), hypertension, dyslipidemia, digestive diseases, and arthritis, and subgroup analyses were performed in all 3 cohorts. As shown in Fig. 3, the association between diabetes mellitus of different durations and hip fracture was not significantly different in all subgroups; all P for interaction > 0.05.

### Sensitivity analysis

After excluding participants who had been involved in a traffic accident or any major accidental injury (*n* = 1,774), a total of 8,153 participants were included, and both univariate and multivariate analyses had similar results as before. Compared with participants without diabetes, participants with established diabetes had a 63% increased risk of hip fracture, HR and 95% CI 1.63 (1.08 ~ 2.47), *P* = 0.021; participants with disease duration ≥ 6 years had a 137% increased risk of hip fracture, HR and 95% CI 2.37 (1.37 ~ 4.1), *P* = 0.002; cohort 3’s HR and 95% CI for the test of trend was 1.16 (1.02 ~ 1.32), *P* = 0.025 (Table 3).

**Table 3:** Association between diabetes and hip fracture after excluding participants who had been involved in a traffic accident or any major accidental injury

Cohorts	*n*. total	Cases (%)	Univariate analysis			Multiplicity analysis	
			HR (95% CI)	*P*-value		HR (95% CI)	*P*-value
Cohort 1							
No diabetes	7157	335 (4.7)	1(Ref)			1(Ref)	
Newly diagnosed diabetes	996	59 (5.9)	1.28 (0.97 ~ 1.69)	0.082		1.17 (0.84 ~ 1.62)	0.353
Cohort 2							
No diabetes	7157	335 (4.7)	1(Ref)			1(Ref)	
Newly diagnosed diabetes	558	28 (5)	1.08 (0.73 ~ 1.59)	0.703		0.82 (0.5 ~ 1.34)	0.431
Known diabetes	438	31 (7.1)	1.54 (1.06 ~ 2.22)	0.022		1.63 (1.08 ~ 2.47)	0.021
Cohort 3							
No diabetes	7157	335 (4.7)	1(Ref)			1(Ref)	
Newly diagnosed diabetes	558	28 (5)	1.08 (0.73 ~ 1.59)	0.703		0.82 (0.5 ~ 1.34)	0.431
Duration 1–3 years	145	9 (6.2)	1.33 (0.69 ~ 2.59)	0.393		1.62 (0.82 ~ 3.18)	0.164
Duration 3–6 years	144	4 (2.8)	0.59 (0.22 ~ 1.58)	0.295		0.76 (0.28 ~ 2.07)	0.593
Duration ≥ 6 years	149	18 (12.1)	2.7 (1.68 ~ 4.34)	< 0.001		2.37 (1.37 ~ 4.1)	0.002
Trend.test	8153	394 (4.8)	1.17 (1.05 ~ 1.31)	0.005		1.16 (1.02 ~ 1.32)	0.025

Multiplicity analysis: adjusted for gender, age, education, marital, residence, smoke, drink, hypertension, dyslipidemia, chronic lung diseases, liver disease, heart problems, stroke, kidney disease, and arthritis

Abbreviations: HR, hazards ratio; CI, confidence interval

## Discussion

In this cohort study, we stratified the participants by no diabetes, newly diagnosed diabetes, and known diabetes and its duration to analyze the association of different durations of diabetes with hip fractures. Compared to participants without diabetes, we arrived at the following: (1) overall diabetes was not significantly associated with hip fracture; (2) newly diagnosed diabetes was also not associated with hip fracture; (3) known diabetes increased the risk of hip fracture by 69%; (4) diabetes with a disease duration of < 6 years did not increase the risk of hip fracture; (5) at a disease duration of ≥ 6 years, the hip fracture risk increased by 120%. These 5 findings did not differ significantly across subgroups of gender, age, hypertension, dyslipidemia, digestive disease, and arthritis status. In sensitivity analyses after excluding participants who had been involved in traffic accidents, the results were similar to the previous ones.

In the studies by Martinez-Laguna D et al. [26], Koh WP et al. [20], and Janghorbani M et al. [22], overall diabetes increased the risk of hip fracture by 20–98%. However, in our study, overall diabetes was not significantly associated with hip fracture. Melton et al. found that diabetes with a duration of < 10 years did not increase the risk of hip fracture, and the standardized incidence of hip fracture was 1.5 times higher when the duration was ≥ 10 years [21]. In our present study, we found that diabetes with a duration of ≥ 6 years was associated with a 120% increased risk of hip fracture, confirming that a longer duration of diabetes is more likely to be associated with hip fractures. The reason for this difference in results may be related to the method of assessing diabetes, as undetected diabetes was not adequately taken into account in the above study.

Unlike the current study, other studies have reported gender differences in the association between diabetes and fractures. Mosenzon O et al. found that females with diabetes had a 92% higher overall fracture risk than males [27]. In another cohort study of older males aged ≥ 70 years [28], it was found that fracture risk was not associated with the presence of diabetes. In a meta-analysis by Moayeri A et al. [29], diabetes was associated with a 20% increased risk of hip fracture, with males suffering from diabetes having a higher relative risk of overall fracture than females. However, in our study, no gender differences were found.

The yearly incidence of hip fracture in our study population was 642/100,000, which is slightly lower than the global average of 681/100,000 in 2019 [13]. In addition, Western Europe and North America have higher hip fracture incidence rates of 1,468/100,000 and 1,084/100,000, respectively [13]. Although previous studies have found that Asian populations have lower bone density than American populations [30], the aforementioned epidemiologic surveys have shown that the absolute risk of hip fracture in Asian populations is lower than in Europe and the United States. Nonetheless, considering that China accounts for more than one-sixth of the world’s population, hip fracture affects a wide range of Chinese populations equally severely. Our study examines the association between the course of diabetes and hip fracture and provides a reliable basis for the prevention of hip fracture in the diabetic population.

The risk of hip fracture in people with diabetes may differ depending on the duration of diabetes, and the reasons for this difference need to be further investigated. Insulin resistance, long-term chronic inflammation, decreased trabecular bone, decreased bone mineral density, and osteoporosis are closely associated with hip fracture, and the effect of diabetes on them is time-dependent [31–36]. Moreover, when bone density is normal or even elevated, diabetes can lead to fracture through altered bone turnover, antidiabetic therapy, and weakening of the body, a condition that can be considered as ‘diabetic osteopathy’ or ‘bone fragility in diabetes’ [16, 31, 37–40], and the association between these factors and the duration of diabetes is unclear.

This study is the first cohort study based on a nationally representative CHARLS to examine the relationship between diabetes duration and hip fracture, and our findings are generalizable to a Chinese middle-aged and older community-based population. Moreover, considering that the awareness rate of diabetes in the Chinese community population is only 36.7% [41], we specifically included newly diagnosed diabetes in our analyses and created multiple stratified cohorts, which made our findings more realistic. However, there were some limitations in our study. Firstly, due to the lack of OGTT 2-hour glucose, this may result in some potentially diabetes patients going undiagnosed. Second, it was not clear in our study whether the participants’ diabetes was type 1 or type 2 because there is no distinction between types of diabetes in CHARLS. However, according to previous epidemiological findings [42, 43], more than 90% of the Chinese diabetes population is type 2 diabetes. Third, due to sample size and missing data limitations, we did not include diabetes treatment modalities among the confounders we included, which may also be associated with hip fracture. Further studies with larger sample sizes and different treatment regimens on the association between diabetes duration and hip fracture are warranted.

## Conclusion

Neither newly diagnosed diabetes nor known diabetes with a disease duration of < 6 years was associated with hip fracture compared with no diabetes patients. When the duration of diabetes is ≥ 6 years, the risk of hip fracture is significantly increased. For people with diabetes, especially middle-aged and older populations with a duration of more than six years, regular bone density testing and fracture risk assessment should be performed as part of public health efforts to identify and intervene in a timely manner for potential fracture risk.

## Data Availability

The data used in our study comes from China Health and Retirement Longitudinal Study (CHARLS), a publicly available database. This data can be found here: https://charls.pku.edu.cn/.

## Abbreviations

CHARLS: China Health and Retirement Longitudinal Study
HbA1c: Glycated hemoglobin A1c
HR: Hazard ratios
CI: Confidence intervals
SD: Standard deviation
IQR: Interquartile range
